# Succus Alterans in Rheumatism and Syphilis

**Published:** 1887-09

**Authors:** 


Succus Alterans in Rheumatism and Syphilis. — We are reliably informed that the preparation Succus Alterans (McDade) is becoming a very popular remedy with the profession, and being very extensively prescribed in general practice as an alterative tonic, aside from its use in syphilitic diseases. The good results from its use in treatment of rheumatism, of chronic character especially, is worthy of consideration. The remedy is certainly growing in favor, and as no great claims have ever been made for it, but simply placed upon its own merit, we think it could possess no higher recommendation. —*Indiana Medical Journal.*

